# From online collaboration to clinical insight: identifying heterotopic ossification through a global hernia surgery network

**DOI:** 10.3389/jaws.2026.16362

**Published:** 2026-07-16

**Authors:** Vanessa Buie, Brian P. Jacob

**Affiliations:** 1 Biological Sciences Division, University of Chicago, Chicago, IL, United States; 2 Icahn School of Medicine at Mount Sinai, New York, NY, United States

**Keywords:** abdominal wall reconstruction, digital scholarship, heterotopic ossification, minimally invasive hernia repair, social media in surgery

## Introduction

I first encountered heterotopic ossification (HO) of the abdominal wall not in the literature, but in clinic.

The patient was several months out from a robotic extended totally extraperitoneal (ETEP) repair for an umbilical extraction-site hernia following robotic prostatectomy, combined with diastasis recti plication. He presented with a new concern: a firm, hard, palpable mass along his midline that he had noticed approximately 4 months after surgery. On examination, this was not scar tissue or a suture granuloma. The mass felt dense, fixed, and distinctly abnormal.

The most plausible explanation I could arrive at was calcified mesh. Yet even that felt unsatisfying. Mesh calcification in this setting seemed unlikely, and the focal, linear nature of the finding did not align with typical mesh-related complications. I reviewed the imaging with a partner, who echoed the same sentiment: *“Strange—but what else could it be?”*

With growing uncertainty, I did what many early-career surgeons do - I called my fellowship mentor. Without hesitation, he responded: *“That’s heterotopic ossification. Search it on the* International Hernia Collaboration.” The International Hernia Collaboration (IHC) is a private Facebook group for surgeons to connect, support one another and advance clinical knowledge in abdominal wall surgery.

Within minutes, I found nine distinct posts, four after minimally invasive repair which described nearly identical presentations, accompanied by over 150 comments from surgeons across the globe. These discussions detailed operative findings, technical pearls, recurrence patterns, and, most importantly, practical management strategies. Surgeons described encountering rigid, bone-like structures along midline repairs after minimally invasive hernia surgery, often mistaken initially for mesh calcification or recurrence.

Only then did I turn to literature. What I found was sparse, dated, and almost entirely rooted in open laparotomy-era surgery. Existing reports described HO almost exclusively after vertical midline incisions, frequently near the xiphoid, and offered little guidance relevant to modern robotic or laparoscopic abdominal wall reconstruction. No management algorithms existed for minimally invasive cases. No discussion addressed barbed sutures, retromuscular planes, or diastasis repair.

It was ultimately the collective experience shared within the IHC that shaped my operative plan, contingency strategies (including having rongeurs available), informed patient counseling, and guided intraoperative teaching for my resident. *“This is bone, and it can be sharp.”*

This experience does not argue for abandoning scientific literature or the academic pursuit of evidence. Rather, it underscores a growing reality in modern surgical practice: surgeon-driven global communities, enabled by social media, function as powerful and underrecognized engines of early clinical discovery. These communities facilitate pattern recognition, identification of rare or emerging complications, and rapid hypothesis generation, often well before traditional publication pathways can respond.

In an era where surgical techniques evolve faster than traditional publication cycles, recognizing the complementary role of collective surgeon experience is not optional, it is essential.

This opinion paper uses heterotopic ossification as a case study to illustrate how social media-enabled, surgeon-led networks contribute meaningfully to modern surgical knowledge. Its purpose is threefold:

1. to synthesize the existing literature on heterotopic ossification in abdominal surgery and highlight its limitations;
2. to draw attention to a novel and previously unreported presentation of HO following minimally invasive abdominal wall reconstruction; and
3. to propose that professional social media–enabled, surgeon-led communities function as accelerators of clinical insight, particularly for rare or emerging complications that fall outside traditional research paradigms.

## When the literature lags clinical reality

Heterotopic ossification (HO) refers to the formation of bone in soft tissues where bone does not normally exist [1]. It is considered a rare complication of abdominal wall surgery, most noted in scar tissue and after large laparotomies associated with trauma and vascular surgery. It may not be as uncommon as is thought with radiographic case series reporting an incidence up to 25% after laparotomy, most commonly vertical and located within the linea alba [2].

Nonetheless, published literature is dominated by isolated case reports or small series that establish HO can occur in abdominal scars, but offer limited insight into risk factors, prevention, or management [1–3]. Most importantly, they provide no framework applicable to modern minimally invasive abdominal wall reconstruction. As a result, contemporary presentations fall outside what the literature prepares surgeons to recognize.

Etiologic theories for heterotopic ossification have traditionally centered on two primary mechanisms: (1) intraoperative seeding of perichondral cells from the xiphoid process and (2) trauma-induced metaplasia of multipotent, undifferentiated mesenchymal connective-tissue cells [1, 4, 5]. In contrast to these historical descriptions, emerging cases of HO following minimally invasive hernia repair occur in the absence of laparotomy incisions, direct bone exposure, or overt surgical trauma (assuming, as is often implied, that minimally invasive surgery is inherently less traumatic).

A third, highly relevant yet comparatively underemphasized hypothesis has been proposed: excessive suture-line tension, which may result in intramuscular implantation and subsequent ossification of periosteal particles avulsed from sites of muscular insertion into bone, with or without associated fascial margin necrosis [6]. This mechanism, later emphasized in radiologic series, offers a plausible explanation for HO observed along suture lines and in non-vertical incisions, including cesarean section scars [2]. Extending this framework to minimally invasive abdominal wall reconstruction necessitates consideration of additional contributory factors, including chronic inflammatory signaling, mechanotransduction, and material–tissue interactions that may collectively create a permissive environment for ectopic bone formation.

The existing literature, while valuable, reflects a surgical era that is no longer inclusive of current surgical modalities. In this case, it confirmed that traditional models were insufficient rather than providing actionable guidance.

## Social media as a global pattern-recognition tool

The recognition of HO after minimally invasive abdominal wall surgery did not originate from traditional academic channels, but from collective surgeon experience shared within a professional social media forum. Through real-time discussion, surgeons across institutions and countries independently described clinical presentations, post operative findings and surgical outcomes. Through asynchronous, real-time discussion, these isolated experiences converged into a recognizable pattern. Surgeons shared operative experiences, described technical considerations and adaptations, discussed recurrence and offered practical management strategies.

What emerged was not anecdote alone, but convergence.

This process illustrates how surgeon-driven communities can function as early warning systems for rare or evolving complications. The real-time aggregation of experience across institutions, geographic regions, and practice settings enables peer consultation and pattern recognition that would otherwise take years to surface through traditional publication cycles.

Importantly, these communities do not generate conclusions, they generate questions. They accelerate hypothesis formation and identify phenomena worthy of formal study, serving as a complementary pathway from observation to evidence.

## Revisiting etiology through collective experience

Viewed through the lens of collective surgeon observation, HO after minimally invasive abdominal wall reconstruction challenges existing etiologic models. The absence of laparotomy incisions and direct bone exposure suggests that alternative mechanisms may be at play. Recurrent themes emerging from online discussions including: tensioned midline closures, barbed suture use, and retromuscular dissection planes, invite reconsideration of how mechanical forces, inflammation, and biomaterial interactions may contribute to ectopic bone formation.

While these observations do not establish causality, they highlight hypothesis that would be difficult to generate from isolated cases alone. The value of the digital forum lies not in replacing scientific validation, but in sharpening the questions that merit rigorous investigation.

## Implications for practice, education, and research

From a clinical standpoint, awareness of HO as a potential complication after minimally invasive abdominal wall surgery has immediate implications for diagnosis, operative planning, and patient counseling. Surgeons must consider HO in the differential diagnosis of postoperative midline masses or unexplained pain and be prepared for unexpected intraoperative findings.

Equally important is the educational value of these cases, which provide opportunities to teach trainees about rare complications, adaptive surgical decision-making, and the limitations of existing evidence when confronting novel presentations.

From a research perspective, early recognition through surgeon networks may represent the first step in identifying phenomena that warrant structured study. Without this initial signal, many rare complications may never reach the threshold required for traditional investigation.

## Rethinking evidence in the digital era

This case underscores the need to broaden how evidence is recognized and generated in contemporary surgery. Social media–enabled surgeon communities do not replace peer-reviewed literature; they complement it. They facilitate early pattern recognition, accelerate hypothesis formation, and identify gaps worthy of formal investigation.

In an era where surgical innovation often outpaces traditional publication cycles, dismissing these platforms risks delaying recognition of clinically meaningful complications and perpetuating knowledge gaps. The pathway from anecdote to evidence is not a leap–it is a process, and digital collaboration increasingly represents its earliest stage.

## Conclusion

Heterotopic ossification following minimally invasive abdominal wall surgery may be a rare complication, but it serves as a case study in how modern surgical knowledge emerges. By integrating collective surgeon experience with rigorous academic inquiry, the surgical community can more rapidly identify, contextualize, and study novel phenomena. Embracing this complementary pathway, from online collaboration to clinical insight, will be essential to advancing patient care in an increasingly connected and rapidly evolving surgical landscape.
